# ILF2 Contributes to Hyperproliferation of Keratinocytes and Skin Inflammation in a KLHDC7B-DT-Dependent Manner in Psoriasis

**DOI:** 10.3389/fgene.2022.890624

**Published:** 2022-05-02

**Authors:** Xiran Yin, Zhenxian Yang, Mingsheng Zhu, Cheng Chen, Shan Huang, Xueqing Li, Hua Zhong, He Wen, Qing Sun, Xiaojing Yu, Jianjun Yan

**Affiliations:** ^1^ Department of Dermatology, Qilu Hospital, Shandong University, Jinan, China; ^2^ Laboratory of Basic Medical Science, Qilu Hospital, Shandong University, Jinan, China; ^3^ Department of Hand and Foot surgery, Shandong Provincial Hospital, Jinan, China

**Keywords:** ILF2, KLHDC7B-DT, psoriasis, proliferation, inflammation

## Abstract

**Background:** The extensive involvement of interleukin enhancer binding factor 2 (ILF2) in RNA stability and the inflammatory response is well documented. Aberrant long noncoding RNA (lncRNA) expression contributes to the pathogenesis of psoriasis. However, little is known about the role of ILF2 in psoriasis.

**Objective:** To investigate the role of ILF2 and KLHDC7B-DT in psoriasis.

**Methods:** LncRNA expression in psoriatic tissues was measured by lncRNA microarray and qRT–PCR. Normal human epidermal keratinocytes (NHEKs), HaCaT cells, and Ker-CT cells stimulated with M5 (IL-17A, IL-22, IL-1α, oncostatin M, and TNF-α) were used to establish a psoriasis model *in vitro*. Fluorescence *in situ* hybridization was used to detect the distribution of KLHDC7B-DT and ILF2 in keratinocytes. The proliferative effects of KLHDC7B-DT and ILF2 on keratinocytes were demonstrated by EdU assay and flow cytometry. ELISA was used to detect the secretion levels of cytokines. RNA pull-down and RNA immunoprecipitation (RIP) were used to detect the direct binding of KLHDC7B-DT with ILF2. Western blotting was used to detect the proteins related to STAT3/JNK signalling pathways.

**Results:** ILF2 and KLHDC7B-DT were significantly overexpressed in psoriatic tissues and M5-induced keratinocytes. KLHDC7B-DT promoted the proliferation of keratinocytes and induced the secretion of IL-6 and IL-8. KLHDC7B-DT could directly bind to ILF2 and activate the STAT3 and JNK signalling pathways. KLHDC7B-DT expression was regulated by ILF2. M5-induced proliferation and inflammatory cytokine secretion in keratinocytes was inhibited after ILF2 knockdown. Furthermore, we found that ILF2 promoted keratinocyte proliferation and the inflammatory response in a KLHDC7B-DT-dependent manner.

**Conclusions:** ILF2 and KLHDC7B-DT are involved in the hyperproliferation of keratinocytes and skin inflammation in psoriasis. In addition, ILF2 functions in a KLHDC7B-DT-dependent manner.

## Introduction

Psoriasis is a common immune-mediated chronic inflammatory skin disease characterized by red, scaly skin plaques (Boehncke and Schön, 2015; Srivastava et al., 2017). Recent studies have shown that immune, genetic and environmental factors are all responsible for the pathogenesis of psoriasis (Nickoloff et al., 2007a; Lin et al., 2011). Accumulating studies have suggested that psoriasis is mainly caused by abnormal crosstalk between keratinocytes and immune cells. Various cytokines secreted by immune cells activate keratinocytes and lead to keratinocyte secretion of proinflammatory cytokines, thereby sustaining the inflammatory response (Albanesi et al., 2005; Albanesi et al., 2007).

Long noncoding RNA (lncRNA) is a noncoding RNA with a length of more than 200 nucleotides and limited protein-encoding abilities (Fatica and Bozzoni, 2014; Iyer et al., 2015). Recent studies have confirmed that some inflammatory diseases, such as psoriasis, rheumatoid arthritis (RA) (Wu et al., 2015), Crohn’s disease (CD) and ulcerative colitis (UC) (Mirza et al., 2015), and multiple sclerosis (MS) (Santoro et al., 2016), show abnormal lncRNA expression. Psoriasis susceptibility-related RNA gene induced by stress (PRINS) was initially identified as a biomarker of genetic susceptibility to psoriasis (Szegedi et al., 2010). Our previous study confirmed that lncRNA MSX2P1 was involved in the pathogenesis of psoriasis by directly binding to miR‐6731‐5p and activating S100A7 (Qiao et al., 2018). These findings demonstrate that differentially expressed lncRNAs contribute to the pathogenesis of psoriasis.

In this study, we performed a lncRNA microarray and found 2,194 differentially expressed lncRNAs (1,123 upregulated and 1,071 downregulated; fold change ≥2 and *p* < 0.05). The microarray results showed that ENST00000609178, also named KLHDC7B-DT (NCBI Gene ID: 105373098), was upregulated (fold change of 17.05) in psoriatic lesions compared to normal tissues. Previous reports have shown that KLHDC7B-DT activates STAT3 signalling by inducing IL-6 secretion (Li et al., 2021). STAT3 signalling and IL-6 are closely related to the pathogenesis of psoriasis, and we hypothesized that KLHDC7B-DT might participate in regulating the inflammatory response and be involved in the pathogenesis of psoriasis.

ILF2, also known as nuclear factor 45 (NF45), is crucial for cell growth and the inflammatory response (Du et al., 2019). Recent studies have shown that ILF2 can promote cell proliferation and the inflammatory response (Jia et al., 2020; Zhang et al., 2021). ILF2 forms a complex with the 90 kDa interleukin enhancer binding factor 3 (NF90, ILF3) (Zhao et al., 2005; Kuwano et al., 2008). A previous study confirmed that lncRNAs could promote cell proliferation through association with the NF45/NF90 complex (Huang et al., 2020). In the present study, we found that both ILF2 and KLHDC7B-DT were overexpressed in psoriatic lesions as well as M5-treated keratinocytes compared with controls. KLHDC7B-DT could directly bind with ILF2, and its expression was regulated by ILF2. As KLHDC7B-DT and ILF2 were significantly overexpressed in psoriatic lesions and closely related to inflammatory responses, KLHDC7B-DT and ILF2 were selected as potential candidates for this study.

Here, we explored the role of ILF2 and KLHDC7B-DT in the pathogenesis of psoriasis for the first time. We verified the effects of ILF2 and KLHDC7B-DT on the proliferation of keratinocytes and the secretion of IL-6 and IL-8. In addition, we showed that KLHDC7B-DT promoted the proliferation of keratinocytes and induced the secretion of IL-6 and IL-8 by activating the STAT3 and JNK signalling pathways. Furthermore, we found that ILF2 functions in a KLHDC7B-DT-dependent manner in psoriasis. Our findings provide a potential therapeutic strategy for psoriasis.

## Materials and Methods

### Patient and Tissue Sample Collection

Ten samples were collected from patients with vulgaris psoriasis (6 males and 4 females; aged 24–49 years) from Qilu Hospital of Shandong University. The patients had not received systemic therapy or topical treatment within 3 months. In addition, 10 normal tissues were collected from healthy volunteers (6 males and 4 females; aged 22–40 years). The healthy subjects had no family history of psoriasis or any other autoimmune diseases. We separated the epidermis from psoriatic lesions and healthy control tissues for further experiments. This study was approved by the Ethics Committee of Shandong University, Qilu Hospital (Jinan, China), and all patients provided written informed consent.

### LncRNA Microarray

The microarray was performed by Shanghai shbio Biotechnology Co., Ltd. The results were detected by an Agilent microarray scanner and analyzed by Agilent feature extraction software.

### Cell Isolation and Culture

Purified normal human epidermal keratinocytes (NHEKs) were obtained after 2–3 generations. HaCaT cells (Procell Life Science & Technology Co., Ltd.) were cultured in Dulbecco’s modified Eagle’s medium (DMEM) (Gibco, United States) containing 10% foetal bovine serum (FBS) (Sangon Biotech, China), 100 μg/ml streptomycin and 100 U/ml penicillin. Ker-CT (CRL4048™) from American Type Culture Collection (ATCC) was cultured in KGMGold™ BulletKit™ (Lonza 00192060). All cells were incubated in a humidified chamber at 37°C with 5% CO_2_.

### RNA Fluorescence *In situ* Hybridization

KLHDC7B-DT probes (5′-CY3-TAACGCTCTTTCAGTCAGGTGTTCCCC, GenePharma) were used for hybridization. DAPI (Servicebio, G1012) was used for nuclear staining.

### Construction of the Psoriatic Cell Model

When cell confluence reached approximately 60–70%, cells were starved in serum-free DMEM for 12 h. Then, M5 (10 ng/ml final concentration; PeproTech), a cocktail of cytokines, was used to treat NHEKs, HaCaT cells, and Ker-CT cells in serum-free DMEM to induce psoriatic inflammation-like conditions for another 24 h. Controls were left untreated.

### Cell Transfection

For gene knockdown, small interfering RNAs (siRNAs) (GenePharma) of KLHDC7B-DT and ILF2 were transferred into HaCaT and Ker-CT cells using Lipofectamine 2000 transfection reagent (Invitrogen, Carlsbad, United States). For gene overexpression, HaCaT and Ker-CT cells were transfected with the eukaryotic expression vector pcDNA3.1 (GenePharma, Supplementary Figure S1) with jet PRIME transfection reagent (Polyplus, United States). The oligonucleotide sequences are shown in Table 1.

**TABLE 1:** Oligonucleotide sequences.

siRNA	Sense (5′→3′)	Antisense (5′→3′)
siKLHDC7B-DT	GCC​CAG​UCA​UUA​UCA​CAU​ATT	UAU​GUG​AUA​AUG​ACU​GGG​CTT
siILF2	GGA​CAU​UUG​AAG​UGC​AAA​UTT	AUU​UGC​ACU​UCA​AAU​GUC​CTT
siNC	UUC​UCC​GAA​CGU​GUC​ACG​UTT	ACG​UGA​CAC​GUU​CGG​AGA​ATT

### qRT–PCR

qRT–PCR was performed as described in our previous experiments (Yan et al., 2019). The relative gene expression was normalized to GAPDH or U6 and calculated using the 2−ΔΔCt method. The primer sequences are summarized in Table 2.

**TABLE 2:** Sequences of primers for qRT–PCR.

RNA	Forward (5′→3′)	Reverse (5′→3′)
LncRNA KLHDC7B-DT	GTT​GCT​AGT​CCT​CCG​CTT​CGC	GCT​GGC​TTG​CCA​CAG​GTT​ATG
ILF2	CCT​TAG​CAG​CCA​TCC​GAC​AT	TTA​GGG​CCA​AAG​GCT​GTC​TG
GAPDH	GCA​CCG​TCA​AGG​CTG​AGA​AC	TGG​TGA​AGA​CGC​CAG​TGG​A
U6	CAG​CAC​ATA​TAC​TAA​AAT​TGG​AAC​G	ACG​AAT​TTG​CGT​GTC​ATC​C

### Western Blotting

Western blotting was performed as described in our previous experiments (Jiang et al., 2017; Zhao et al., 2017). The following primary antibodies were used in this study: GAPDH, p-JNK1/2, JNK1/2, p-STAT3, STAT3 (all from Cell Signaling Technology, Boston, United States), cyclin D1, BCL-xL (both from Abcam, Cambridge, United Kingdom), and ILF2 (from ZenBioScience, China).

### EdU Incorporation Assay

The EdU (5-ethynyl-2′-deoxyuridine) incorporation assay was performed using a Cell-Light EdU DNA Cell Proliferation Kit (RiboBio, Guangzhou, China).

### Flow Cytometry Analysis

Cell cycle and apoptosis analyses were performed by flow cytometry using a DNA content quantitation assay kit (Solarbio, Beijing, China) and an Annexin V-fluorescein isothiocyanate (FITC) apoptosis measurement kit (BD Biosciences, United States).

### Enzyme-Linked Immunosorbent Assay

After 24 h of transfection as described above, cells were stimulated with M5, and culture supernatants were collected after 12 h. The secretion of IL-6 and IL-8 was measured using specific ELISA kits (Elabscience, China). The absorbance at 450 nm was measured with a microplate reader (BioTek, United States).

### RNA Pull-Down Assays and Mass Spectrometry Analyses

The interaction between KLHDC7B-DT and RNA-binding protein was detected by a Pierce Magnetic RNA–Protein Pull-Down Kit (Thermo Fisher Scientific, United States). A Fast Digest Hind III Kit (Thermo Scientific) and MEGA Script Kit (Life Technologies) were used for digestion, linearization, and transcription. Biotin-labelled KLHDC7B-DT was incubated with total cell lysates of HaCaT cells, and eluted proteins were purified. The sequences of biotin-labelled KLHDC7B-DT are shown in Supplementary Table S1. Finally, interacting proteins were identified by mass spectrometry and Western blotting.

### RNA Immunoprecipitation Assay

RIP was carried out using a Magna RIP Kit (Millipore, Billerica, MA, United States). HaCaT and Ker­CT cells were lysed with RNA lysis buffer, and cell lysates were incubated with RIP buffer containing magnetic beads conjugated to anti-ILF2 (ZenBioScience, #382994) or negative control IgG antibody (Millipore, Billerica, MA, United States) for 4 h at 4°C. Then, qRT–PCR and agarose electrophoresis were implemented to detect RNA. The sequences of primers for qRT–PCR are shown in Supplementary Table S2.

### Immunohistochemistry

IHC was performed as previously described (Jiang et al., 2017; Zhao et al., 2017). Primary antibodies against ILF2 (ZenBioScience, #382994) were used in this study. IHC staining was quantified using H-scores, which incorporate the staining intensity and the percentage of positively-stained cells (range 0–100%).

### Immunofluorescence

Immunofluorescence was performed according to the manufacturer’s directions. Samples were analysed using a Zeiss LSM710 Confocal Laser Scanning Microscope (Carl Zeiss, Oberkochen, Germany).

### Statistical Analysis

Data from at least three independent experiments are presented as the means ± standard deviation (SD). Statistical significance was analysed using GraphPad Prism (version 6, San Diego, United States). Differences between the two groups were analysed using Student’s t test. *p* < 0.05 was considered statistically significant.

## Results

### KLHDC7B-DT Is Upregulated in Psoriatic Tissues and M5-Induced Keratinocytes

**FIGURE 1:**
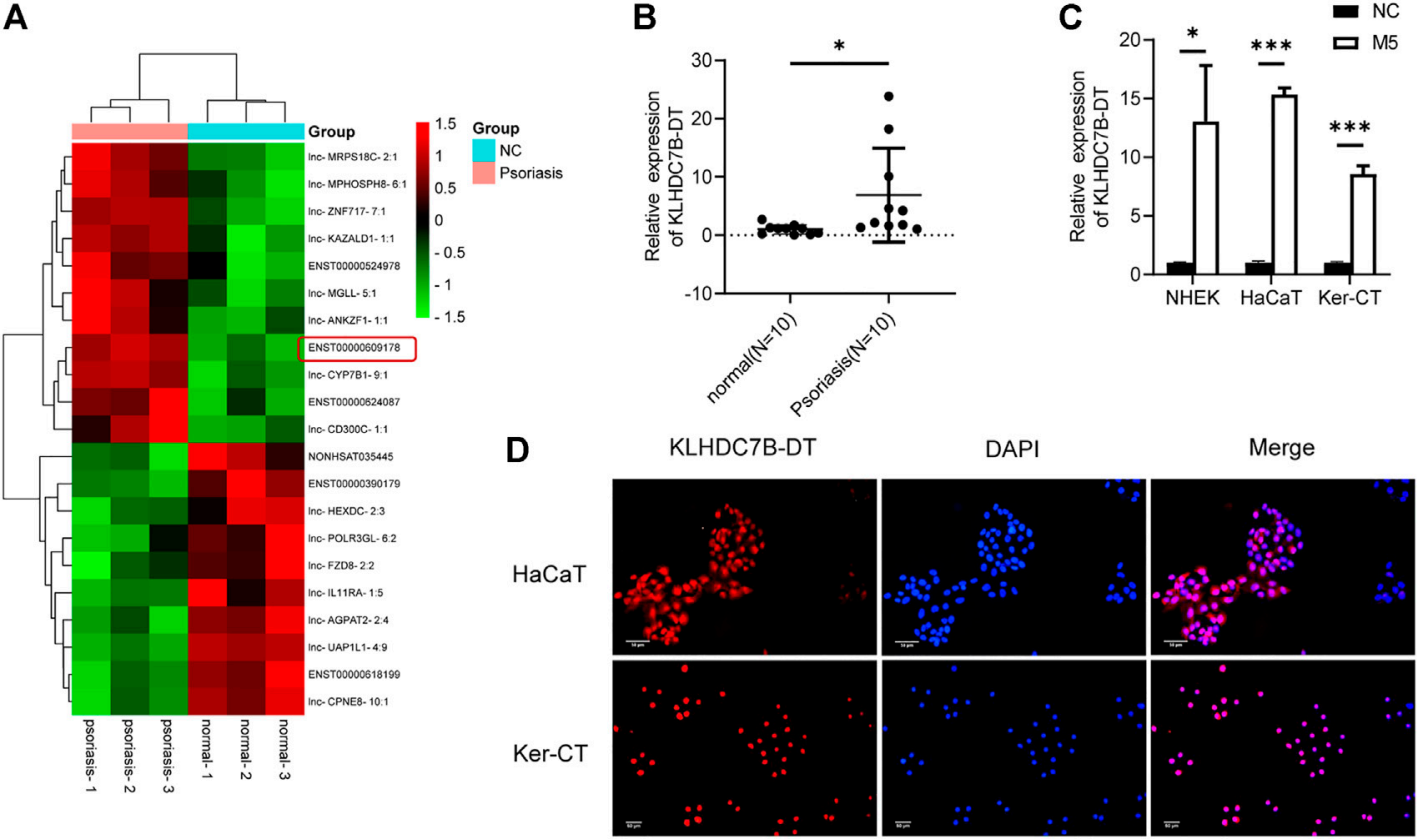
KLHDC7B-DT is upregulated in psoriatic tissues and M5-induced keratinocytes. **(A)** Differentially expressed lncRNAs detected in psoriatic and normal skin tissues using a lncRNA microarray (*n* = 3, Accession: GSE181318). **(B)** The expression level of KLDHC7B-DT in psoriatic tissues and normal controls (*n* = 10). **(C)** The expression level of KLDHC7B-DT in M5-induced NHEKs, HaCaT cells, and Ker-CT cells. **(D)** Fluorescence *in situ* hybridization showed the expression and localization of KLDHC7B-DT in HaCaT and Ker-CT cells. A Cy3-labelled probe (red) was used to show KLDHC7B-DT. DAPI (blue) was performed to show the nuclei of cells. Data are shown as the means ± SD, *p < 0.05, ***p < 0.001. All experiments were repeated at least three times. NHEK: normal human epidermal keratinocytes.

To identify new lncRNAs associated with the pathogenesis of psoriasis, a lncRNA microarray was performed (*n* = 3, accession number: GSE181318). The microarray results showed that KLHDC7B-DT was one of the most significantly upregulated lncRNAs in psoriatic tissues, which was also confirmed by qRT–PCR (Figures 1A,B). Subsequently, an *in vitro* psoriasis model was established by stimulating keratinocytes with M5. As expected, the expression of KLHDC7B-DT was increased in M5-treated NHEKs, HaCaT cells, and Ker-CT cells (Figure 1C). FISH assays in HaCaT and Ker-CT cells indicated that KLHDC7B-DT was mainly distributed in the nucleus (Figure 1D). In addition, Sanger sequencing showed that the sequence of the amplified product was consistent with that of the template (Supplementary Figure S2). These results indicated that aberrantly upregulated KLHDC7B-DT expression might be associated with the pathogenesis of psoriasis.

### KLHDC7B-DT Regulates Proliferation and Inflammatory Factor Secretion in M5-Induced Keratinocytes

**FIGURE 2:**
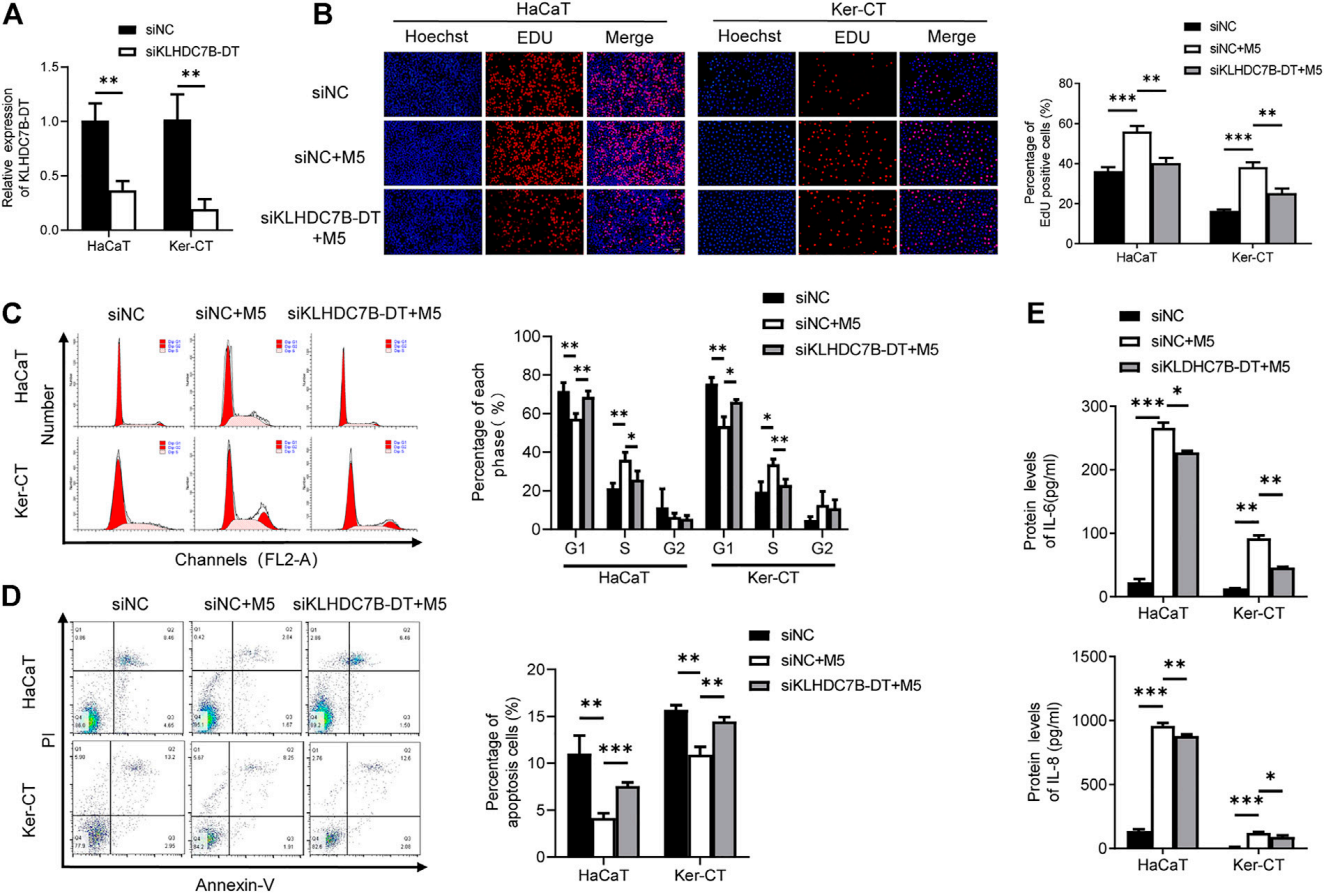
KLHDC7B-DT regulates proliferation and inflammatory factor secretion in M5-induced keratinocytes. **(A)** The efficiency of KLDHC7B-DT knockdown was detected by qRT–PCR. **(B)** Proliferative effects were detected by EdU assay. **(C)** The cell cycle was detected by flow cytometry through PI staining. **(D)** Cell apoptosis was detected by flow cytometry through Annexin-V/PI staining. **(E)** Protein levels of IL-6 and IL-8 were detected by ELISA. Data are shown as the means ± SD, *p < 0.05, **p < 0.01, ***p < 0.001. All experiments were repeated at least three times.

We next investigated the potential biological roles of KLHDC7B-DT in M5-induced keratinocytes. qRT–PCR showed that KLHDC7B-DT expression was significantly suppressed by siRNA in HaCaT and Ker-CT cells (Figure 2A). EdU incorporation assays showed that M5 treatment increased EdU-positive cells, and the proliferation-promoting effect was inhibited by KLHDC7B-DT knockdown (Figure 2B). Cell cycle results indicated that M5 treatment led to a reduced proportion in the G1 phase and an increased proportion in the S phase, and KLHDC7B-DT knockdown reversed the M5-induced effects (Figure 2C). Moreover, cell apoptosis analysis revealed that M5 treatment suppressed the proportion of early and late apoptotic cells, whereas KLHDC7B-DT knockdown induced a higher proportion of early and late apoptotic cells (Figure 2D). These findings suggested that KLHDC7B-DT regulates the proliferation of keratinocytes in psoriasis.

ELISAs showed that the M5-induced expression of IL-6 and IL-8 was partially reversed after KLHDC7B-DT knockdown in keratinocytes (Figure 2E). Collectively, our data demonstrated that KLHDC7B-DT knockdown reverses M5-induced hyperproliferation and the inflammatory response in keratinocytes, indicating that KLHDC7B-DT might play an important role in psoriasis by regulating the proliferation and inflammatory response of keratinocytes.

### Overexpression of KLHDC7B-DT Facilitates Proliferation and Secretion of Inflammatory Factors in Keratinocytes

**FIGURE 3:**
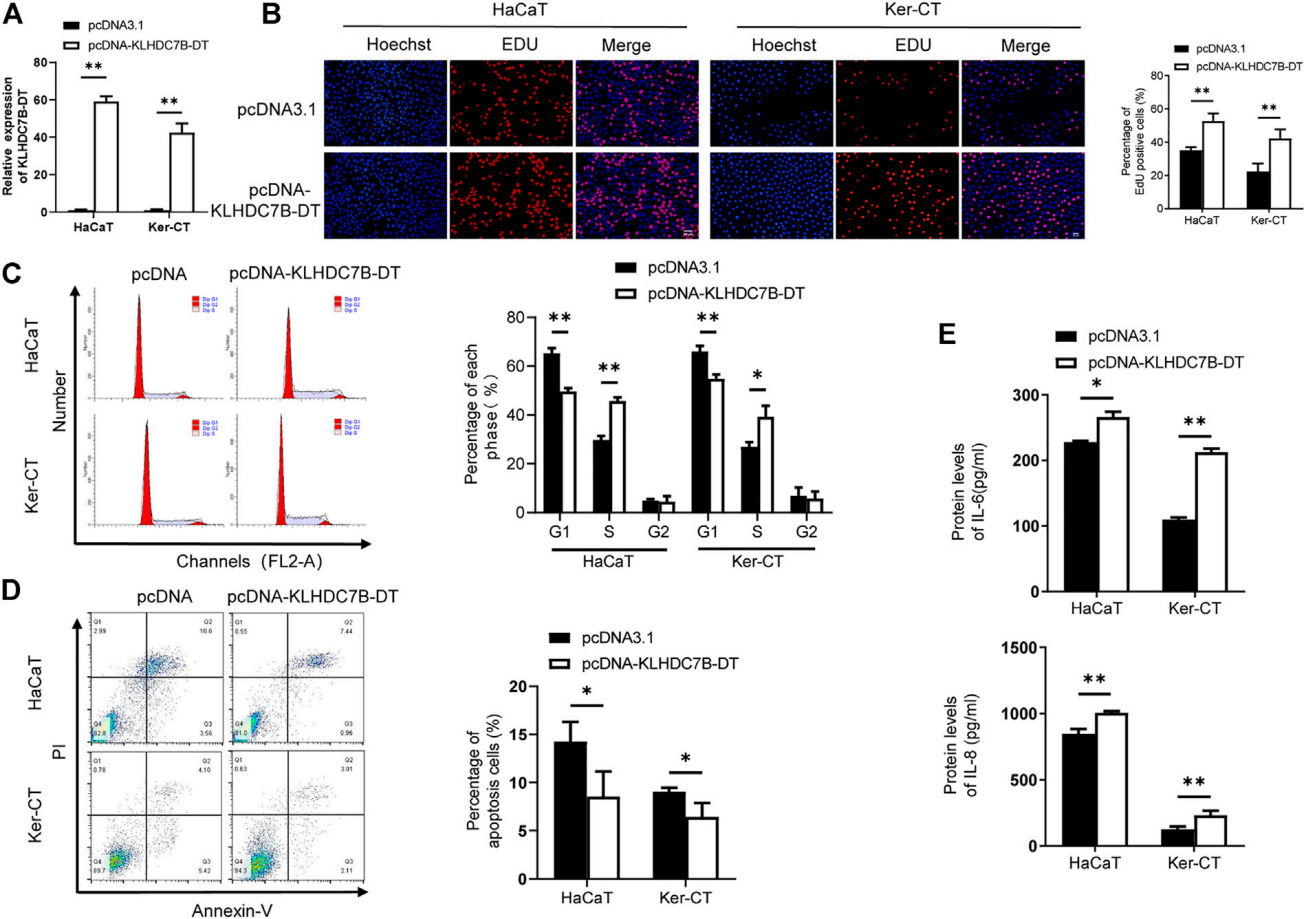
The proliferative and inflammatory effects of KLDHC7B-DT overexpression in M5-induced keratinocytes. **(A)** The efficiency of KLDHC7B-DT overexpression detected by qRT–PCR. **(B)** Proliferative effects of KLDHC7B-DT overexpression on M5-treated keratinocytes detected by EdU assay. **(C)** The cell cycle was detected by flow cytometry through PI staining. **(D)** Cell apoptosis was detected by flow cytometry through Annexin-V/PI staining. **(E)** Protein levels of IL-6 and IL-8 were detected by ELISA. Data are shown as the means ± SD, *p < 0.05, **p < 0.01, ***p < 0.001. All experiments were repeated at least three times.

To further verify the role of KLHDC7B-DT in psoriasis, we stably overexpressed KLHDC7B-DT *via* transfection of a plasmid into M5-induced HaCaT and Ker-CT cells (Figure 3A). EdU assays showed that HaCaT and Ker-CT cells overexpressing KLHDC7B-DT had more EdU-positive cells (Figure 3B). The cell cycle results demonstrated that KLHDC7B-DT overexpression led to a reduced proportion of cells in the G1 phase and an increased proportion of cells in the S phase (Figure 3C). Moreover, cell apoptosis analysis revealed that KLHDC7B-DT overexpression inhibited the proportion of early and late apoptosis (Figure 3D). Overexpression of KLHDC7B-DT also significantly upregulated the IL-6 and IL-8 protein expression levels in M5-treated HaCaT and Ker-CT cells (Figure 3E). Taken together, these findings demonstrated that KLHDC7B-DT overexpression promotes the proliferation and inflammatory response in M5-induced keratinocytes.

### KLHDC7B-DT Binds With the ILF2 Protein and Activates JNK/STAT3 Signalling

**FIGURE 4:**
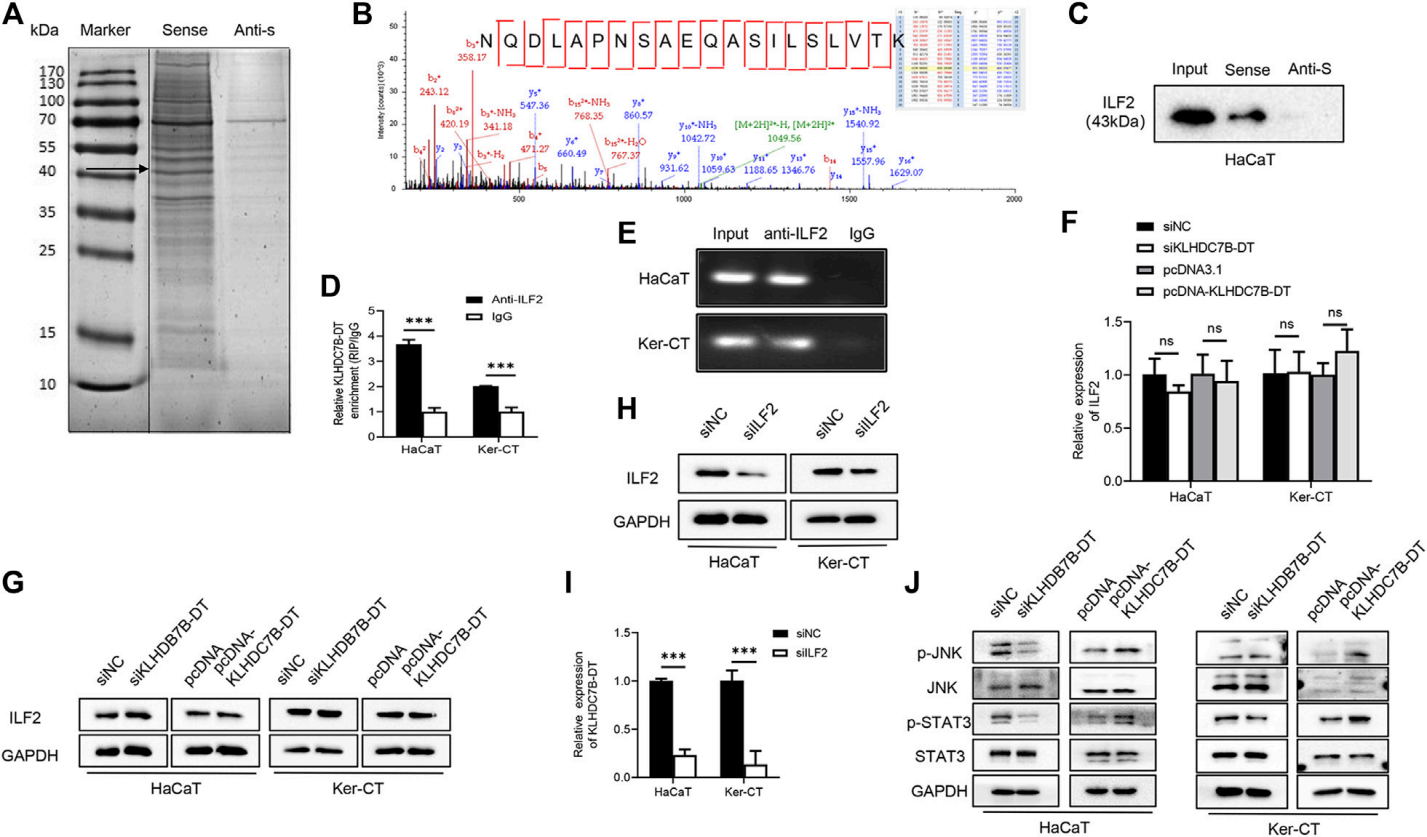
KLHDC7B-DT can bind with ILF2 protein and activate JNK/STAT3 signalling. **(A)** Silver staining of interacting proteins after the KLDHC7B-DT pulldown assay. **(B)** Mass spectrometry of ILF2. **(C)** Western blotting was used to detect ILF2 in the RNA pulldown product. **(D)** RIP experiments were performed using an antibody against ILF2 on lysates from HaCaT and Ker-CT cells; IgG was used as the control group. **(E)** Agarose electrophoresis detected KLDHC7B-DT in products from RIP. **(F,G)** mRNA and protein levels of ILF2 in KLDHC7B-DT knockdown or overexpression keratinocytes. **(H)** The efficiency of ILF2 knockdown was detected by Western blotting. **(I)** The mRNA levels of KLDHC7B-DT after ILF2 knockdown. **(J)** Activation of the STAT/JNK pathway after KLDHC7B-DT knockdown or overexpression. Data are shown as the means ± SD, ***p < 0.001. All experiments were repeated at least three times.

To further understand the molecular mechanism by which KLHDC7B-DT regulates proliferation and the inflammatory response in M5-induced keratinocytes, we precipitated synthesized KLHDC7B-DT *in vitro* and subjected it to mass spectrometric identification. RNA pull-down assays in HaCaT cells showed that several proteins were identified as interacting partners of KLHDC7B-DT (Figure 4A). The binding proteins were further detected and analysed using mass spectrometry, which showed that KLHDC7B-DT could bind to the interacting proteins. Kyoto Encyclopedia of Genes and Genomes analysis suggested that these proteins were involved in the JAK-STAT and MAPK signalling pathways, while Gene Ontology analysis revealed the cell cycle, apoptosis, and inflammation functions of these proteins (Supplementary Figure S3A,B). Based on the mass spectrum assay (Figure 4B), we found that ILF2 was enriched in the KLHDC7B-DT-sense group among the detected proteins. Western blotting validated the existence of ILF2 within the RNA pull-down samples of KLHDC7B-DT in HaCaT cells (Figure 4C). In addition, RIP assays demonstrated the interaction between ILF2 and KLHDC7B-DT in HaCaT and Ker-CT cells (Figure 4D). By detecting the enriched RNA, the amplification band of KLHDC7B-DT was obtained by agarose electrophoresis (Figure 4E).

The expression of ILF2 was detected after KLHDC7B-DT knockdown and overexpression in HaCaT and Ker-CT cells, and no significant differences in ILF2 mRNA or protein expression were found (Figures 4F,G). To further determine whether ILF2 was involved in the upregulation of KLHDC7B-DT in psoriasis, ILF2 was knocked down in HaCaT and Ker-CT cells. The interference efficiency was verified by qRT–PCR (Supplementary Figure S4) and Western blotting (Figure 4H). Notably, ILF2 knockdown inhibited the expression of KLHDC7B-DT (Figure 4I).

Western blotting showed that the levels of p-STAT3 and p-JNK were significantly decreased after KLHDC7B-DT knockdown. In contrast, the protein levels of p-STAT3 and p-JNK were significantly increased after KLHDC7B-DT overexpression (Figure 4J). These results suggested that KLHDC7B-DT functioned by regulating the STAT3 and JNK signalling pathways in psoriasis.

### ILF2 is Upregulated in Psoriatic Tissues and Contributes to the Proliferation and Secretion of Inflammatory Factors in Keratinocytes

**FIGURE 5:**
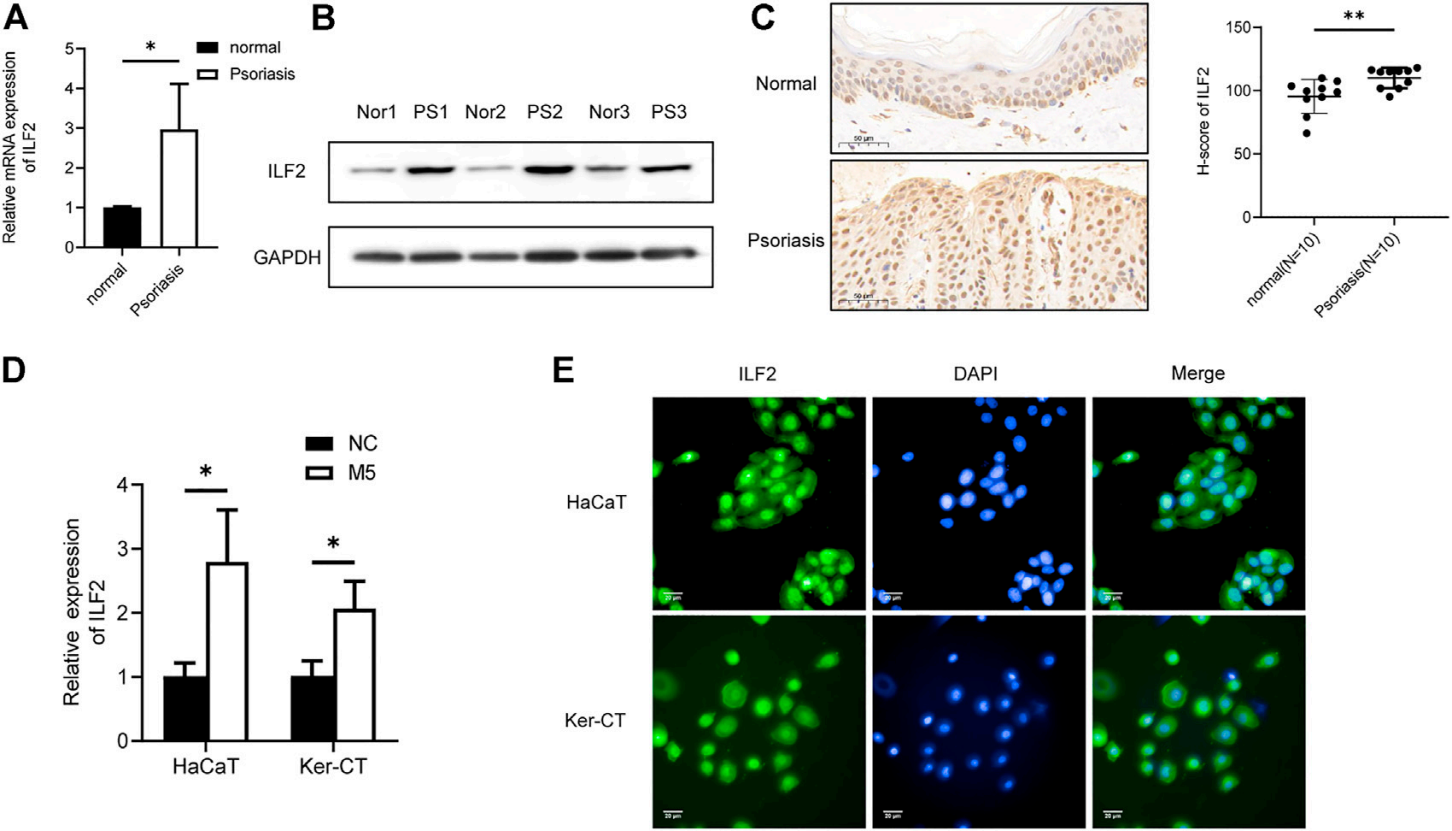
ILF2 is highly expressed in psoriatic lesions. **(A)** The mRNA expression level of ILF2 in psoriatic tissues (*n* = 3). **(B)** The protein expression level of ILF2 in psoriatic tissues (*n* = 3). **(C)** The expression of ILF2 in normal and psoriatic tissues was detected by IHC (*n* = 10). The H-score was calculated based on the percentage of ILF2+ cells and staining intensity.**(D)** The expression level of ILF2 in M5-treated HaCaT and Ker-CT cells. **(E)** Subcellular localization of ILF2 in HaCaT and Ker-CT cells. Data are shown as the means ± SD, *p < 0.05. All experiments were repeated at least three times.

**FIGURE 6:**
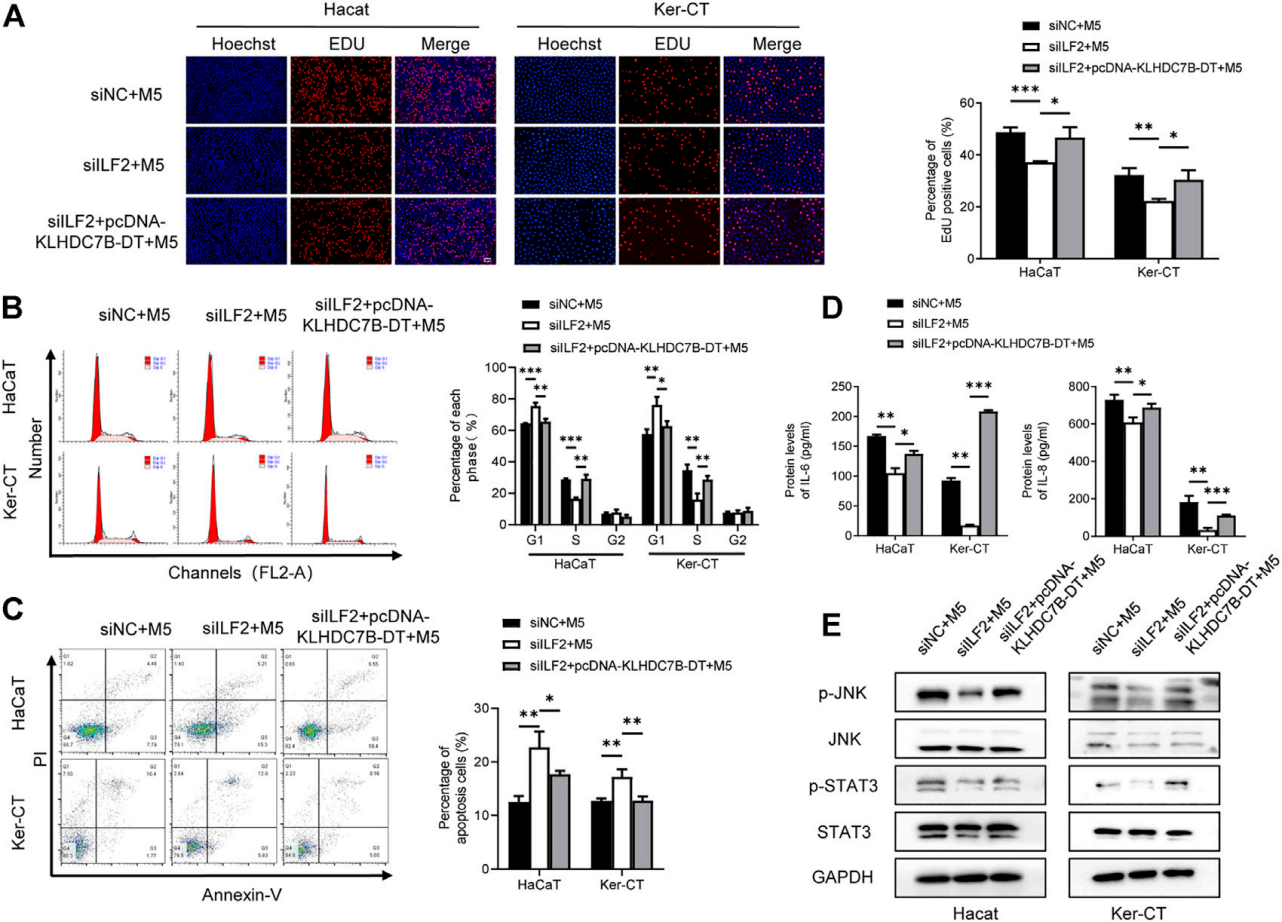
ILF2 promotes hyperproliferation and inflammation through STAT3/JNK pathways in M5-induced keratinocytes. **(A)** Using an EdU assay, the proliferative effects of M5-induced keratinocytes were detected after transfection with siILF2 and siILF2+pcDNA-KLDHC7B-DT. **(B)** The cell cycle was detected by flow cytometry through PI staining. **(C)** Cell apoptosis was detected by flow cytometry through Annexin-V/PI staining. **(D)** Protein levels of IL-6 and IL-8 were detected by ELISA. **(E)** Activation of the STAT/JNK pathway detected by Western blotting. Data are shown as the means ± SD, *p < 0.05, **p < 0.01, ***p < 0.001. All experiments were repeated at least three times.

Next, we investigated the role of ILF2 in psoriasis. qRT–PCR (*n* = 3) and Western blotting (*n* = 3) demonstrated that ILF2 was overexpressed in psoriatic tissues (Figures 5A,B). We carried out IHC staining to analyze the protein expression of ILF2 in 10-paired psoriatic tissues and normal tissues. The H-score indicated that ILF2 expression was increased in psoriatic tissues in contrast to normal tissues (Figure 5C). The expression of ILF2 was increased in M5-treated HaCaT and Ker-CT cells (Figure 1D). A cell immunofluorescence assay demonstrated that ILF2 was mainly localized in the nucleus of HaCaT cells and Ker-CT cells (Figure 5D). EdU assays showed that ILF2 knockdown inhibited the proliferation of keratinocytes (Figure 6A). Cell cycle analysis showed that ILF2 knockdown increased the proportion of the G1 phase and decreased the proportion of the S phase in keratinocytes (Figure 6B). Annexin-V/PI staining results demonstrated that the proportion of apoptotic cells increased after ILF2 knockdown (Figure 6C). In addition, ELISA analysis showed that the increased protein levels of IL-6 and IL-8 in M5-induced keratinocytes were significantly decreased after ILF2 knockdown (Figure 6D). Collectively, these findings showed that ILF2 knockdown inhibits the proliferation, cell cycle and secretion of inflammatory factors and promotes apoptosis in M5-induced keratinocytes.

### ILF2 Promotes Hyperproliferation and Inflammation Through STAT3/JNK Pathways in a KLHDC7B-DT-Dependent Manner

To explore the regulatory mechanism of ILF2 and KLHDC7B-DT in psoriasis, ILF2 siRNA was transfected alone or together with pcDNA-KLHDC7B-DT into M5-treated HaCaT and Ker-CT cells. Then, the proliferation, cell cycle, apoptosis, and inflammatory factor secretion of keratinocytes were detected. The reduced percentage of EdU-positive cells and G1/S growth arrest caused by ILF2 knockdown could be rescued by KLHDC7B-DT overexpression (Figures 6A,B). ILF2 knockdown increased the proportion of apoptotic cells, which was significantly reversed by overexpression of KLHDC7B-DT (Figure 6C). Moreover, the ILF2 knockdown-mediated decrease in IL-6 and IL-8 protein levels was significantly reversed by KLHDC7B-DT overexpression (Figure 6D).

Then, we stably overexpressed ILF2 *via* transfection of a plasmid into M5-induced HaCaT cells. The overexpression efficiency was verified by Western blotting (Supplementary Figure S5). Then pcDNA-ILF2 was transfected alone or together with siKLHDC7B-DT into M5-treated HaCaT cells, the proliferation, cell cycle, apoptosis, and inflammatory factor secretion of keratinocytes were detected. The increased percentage of EdU-positive cells and promotion of G1/S transition caused by ILF2 overexpression could be reversed by KLHDC7B-DT knockdown (Supplementary Figure S6A,B). ILF2 overexpression decreased the proportion of apoptotic cells, which was significantly rescued by KLHDC7B-DT knockdown (Supplementary Figure S6C). Moreover, the ILF2 overexpression-mediated increasein IL-6 and IL-8 protein levels was significantly reversed by KLHDC7B-DT knockdown (Supplementary Figure S6D).

As it has been reported that ILF2 is involved in inflammatory responses through the STAT3 and JNK signalling pathways, we investigated whether ILF2 induces phosphorylation of STAT3/JNK in HaCaT and Ker-CT cells. Interestingly, ILF2 knockdown decreased the phosphorylation levels of STAT3/JNK. More importantly, decreased STAT3/JNK phosphorylation after ILF2 knockdown could be reversed by KLHDC7B-DT overexpression (Figure 6E). Taken together, the results indicated that ILF2 promotes hyperproliferation and inflammation through STAT3/JNK pathways in a KLHDC7B-DT-dependent manner.

## Discussion

Psoriasis is a chronic inflammatory skin disorder (Nickoloff et al., 2007b; Bos, 2007; Boehncke and Sterry, 2009). Keratinocytes actively participate in the initiation and maintenance of psoriatic skin inflammation through the production of various proinflammatory cytokines (Lowes et al., 2014). In this study, we performed a lncRNA microarray, and the results showed that KLHDC7B-DT was one of the most significantly upregulated lncRNAs in psoriatic tissues. Then, we verified the results in psoriatic lesions and a psoriasis model *in vitro* by stimulating keratinocytes with IL-17A, IL-22, IL-1α, oncostatin M, and TNF-α (M5) (Rabeony et al., 2014; Li et al., 2019). We found that the expression levels of KLHDC7B-DT, as well as its binding protein ILF2, were upregulated in both skin lesions and psoriasis model keratinocytes, suggesting that KLHDC7B-DT and ILF2 might be involved in the pathogenesis of psoriasis.

LncRNAs have been revealed to participate in the pathogenesis of psoriasis, including MEG3, GAS5, and MIR31HG (Gao et al., 2018; Jia et al., 2019; Ahmed Shehata et al., 2021). Here, we demonstrated that KLHDC7B-DT positively regulates the proliferation of keratinocytes. Cell cycle analysis and Annexin-V/PI staining indicated that KLHDC7B-DT regulates the G1/S transition and cell apoptosis. These results suggested that KLHDC7B-DT might be involved in the pathogenesis of psoriasis by regulating keratinocyte proliferation and apoptosis.

IL-6 and IL-8 have been proven to play important roles in psoriasis (Elder et al., 1992; Yoshinaga et al., 1995). Previous studies also demonstrated that serum levels of proinflammatory cytokines reflect disease activity and treatment response (Grossman et al., 1989; Biasi et al., 1998). In this study, we found that the expression of IL-6 and IL-8 was regulated by KLHDC7B-DT in keratinocytes. Our findings suggested that KLHDC7B-DT contributes to psoriatic skin inflammation by regulating the expression of IL-6 and IL-8.

It has become increasingly clear that the biological functions of lncRNAs are associated with their unique subcellular localization (Carlevaro-Fita and Johnson, 2019). LncRNAs can function by interacting with proteins *via* structural interactions and/or complementary base pairing (Tang et al., 2020). In this study, both ILF2 and KLHDC7B-DT were found to be mainly distributed in the nucleus. Furthermore, we confirmed the interaction between KLHDC7B-DT and ILF2 protein by RNA pull-down and RIP assays. We found that ILF2 acted as the upstream regulator of KLHDC7B-DT and positively regulated its expression. In addition, previous studies reported that ILF2 was involved in inflammatory responses through the STAT3 and JNK inflammatory signalling pathways (Jia et al., 2020). We confirmed that ILF2 was upregulated in psoriatic tissues and M5-induced keratinocytes. Moreover, overexpression of KLHDC7B-DT rescued the effects of ILF2 knockdown on keratinocyte proliferation and cytokine secretion. In contrast, KLHDC7B-DT konckdown reversed the effects of ILF2 overexpression. These results suggested that ILF2 regulates proliferation and the inflammatory response *via* KLHDC7B-DT in keratinocytes.

Furthermore, KEGG and GO analyses showed that proteins binding with KLDHC7B-DT were involved in the JAK-STAT and MAPK signalling pathways. Previous studies have reported that STAT3 and JNK play crucial roles in regulating immune responses and participate in the pathogenesis of psoriasis (Sano et al., 2005; Liang et al., 2019). IL-6 and IL-8 have been proven to play important roles in psoriasis, and both of them can initiate STAT3 and JNK MAPK pathway, thus involving in regulating cell inflammation and proliferation of keratinocytes (Mori et al., 2011; Chen et al., 2015; Fu et al., 2015). Furthermore, KLHDC7B-DT was found to directly bind to the IL-6 promoter activate IL-6 transcription, up-regulate IL-6 expression. Through enhancing IL-6 secretion, KLHDC7B-DT activated STAT3 signaling in PDAC cells (Jia et al., 2020). In addition to the STAT3 signaling pathway, IL-6 also activates the MAPK cascade (Neurath and Finotto, 2011). Our findings showed that KLHC7B-DT is closely related to the activation of the STAT3 and JNK signalling pathways, and indicated that KLHDC7B-DT activated STAT3 and JNK signaling pathways by promoting IL-6 and IL-8 secretion. Furthermore, overexpression of KLHDC7B-DT rescued the expression of p-STAT3 and p-JNK after ILF2 knockdown, which indicated that ILF2 regulates the STAT3/JNK pathways *via* KLHDC7B-DT. To sum up, our results suggested that ILF2 regulates proliferation and inflammatory reactions *via* the KLHDC7B-DT and STAT3/JNK signalling pathways in keratinocytes.

Although various studies have emphasized the important role of lncRNAs, few have focused on the underlying mechanism driving the abnormal expression of lncRNAs in psoriasis. In the present study, our data demonstrated that upregulated expression of KLHDC7B-DT was associated with high levels of ILF2 in psoriatic tissues.

However, this study was subject to some limitations. Our sample sizes were relatively small, and further investigations with larger samples are required to verify our findings. The expression of KLHDC7B-DT and ILF2 was focused only on psoriasis. Other inflammatory diseases, including atopic dermatitis, should be included to investigate the specificity of the biomarkers.

## Conclusion

In conclusion, we demonstrated that KLHDC7B-DT and ILF2 are overexpressed in psoriatic skin lesions. Overexpression of KLHDC7B-DT in psoriasis was related to the aberrant expression of ILF2. ILF2 and KLHDC7B-DT were involved in the hyperproliferation of keratinocytes and skin inflammation in psoriasis. In addition, we verified that ILF2 functions in a KLHDC7B-DT-dependent manner.

## Data Availability

The datasets presented in this study can be found in online repositories. The name of the repository and accession number can be found below: GEO, NCBI; GSE181318.
