# Supplementary material for: ILF2 Contributes to Hyperproliferation of Keratinocytes and Skin Inflammation in a KLHDC7B-DT-Dependent Manner in Psoriasis
Source: Front Genet. 2022 May 2;13:890624. doi: 10.3389/fgene.2022.890624 (PMC9110045; doi:10.3389/fgene.2022.890624)
Supplement: Supplementary file 1 [file DataSheet1.PDF]

Supplementary Material

Supplementary Figures

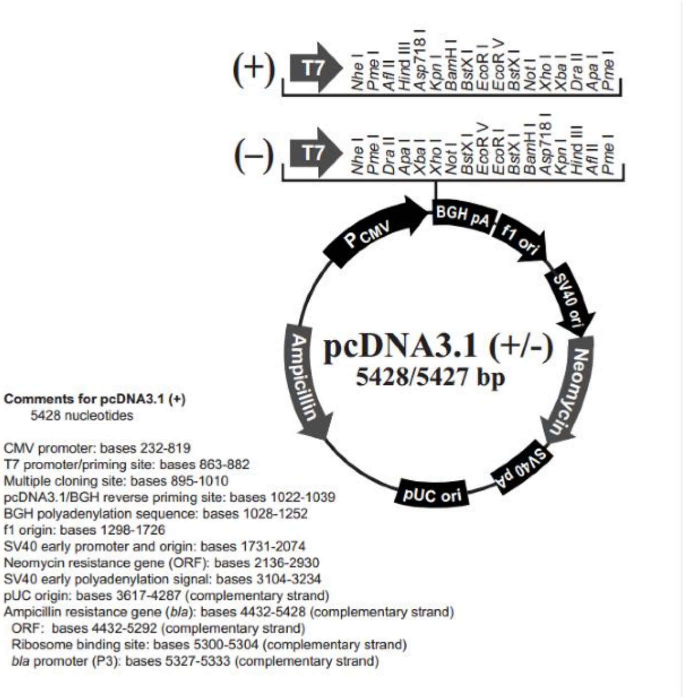

Supplementary Figure 1. The structure of Overexpression plasmid

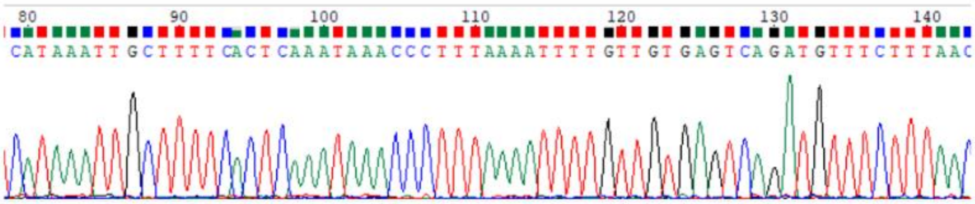

Supplementary Figure 2. Sanger sequencing and full-length sequence of the amplified KLHDC7B-DT were consistent with the template

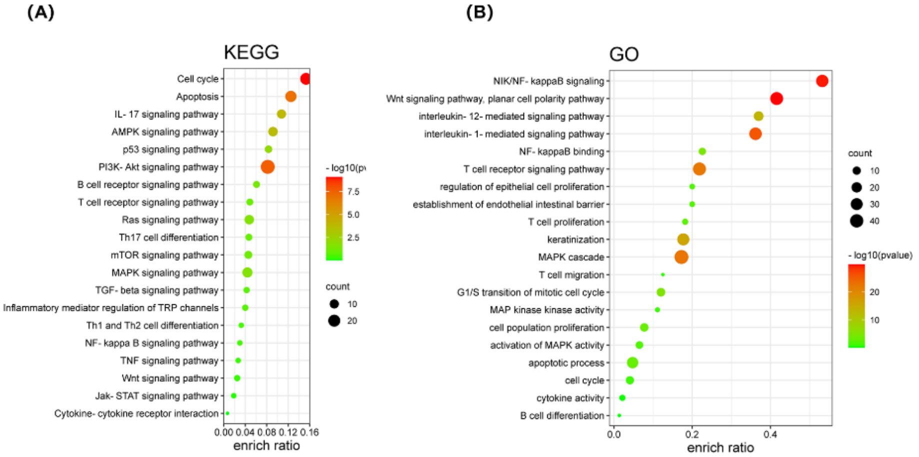

Supplementary Figure 3. (a) Kyoto Encyclopedia of Genes and Genomes analysis of the binding proteins. **(b)** Gene Ontology analysis of the binding proteins.

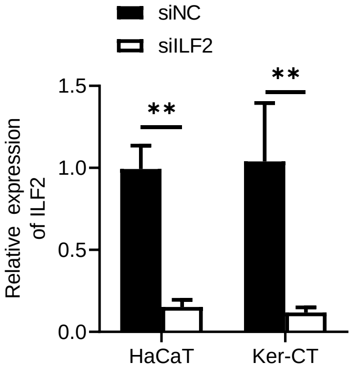

Supplementary Figure 4. The efficiency of ILF2 knockdown was detected by qRT-PCR

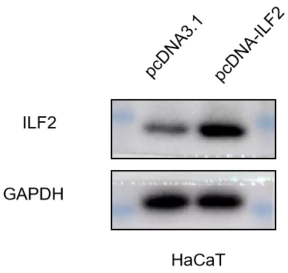

Supplementary Figure 5. The overexpression efficiency of ILF2 was detected by Western blotting

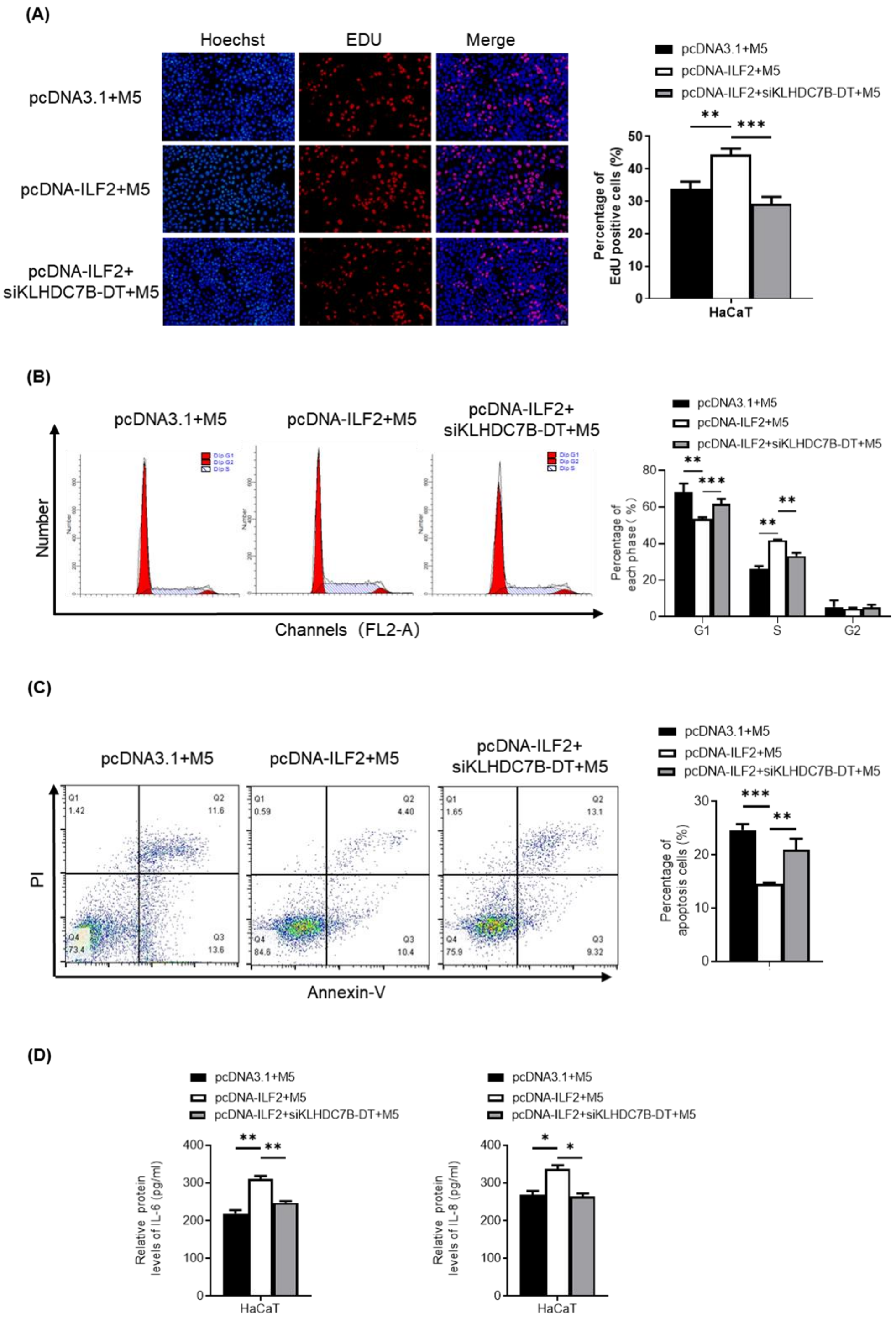

Supplementary Figure 6. ILF2 regulates proliferation and the inflammatory response via KLHDC7B-DT in HaCaT cells. (a) Using an EdU assay, the proliferative effects of M5-induced HaCaT cells were detected after transfection with pcDNA-ILF2 and pcDNA-ILF2+siKLHDC7B-DT. (b) The cell cycle was detected by flow cytometry through PI staining. (c) Cell apoptosis was detected by flow cytometry through Annexin-V/PI staining. (d) Protein levels of IL-6 and IL-8 were detected by ELISA. Data are shown as the means ± SD, *P< 0.05, ** P<0.01, ***P<0.001. All experiments were repeated at least three times.

Supplementary Tables

Supplementary Table 1. Sequences of Biotin-labeled KLHDC7B-DT for RNA pull down assays

Sequences of Sense：
AAGCTTTTCTTAGGCGTCCTCATCCCTTTTCACCTCGGAGCTCAGCGTCTTCCTCAGCAGC
ACTTCCATGTCATCTGCCCCGTGAAATCAGCCTAACGCCGTTTCTCAATGACGTGGATCG
CCCTAGGCCACCGCAACCTTCCGGAAGCTCTCTCAGCTCAGTTCCCATTCTCCCACCATC
TCTTGGTTCTCCTTCTACCTCACCGGTTGCTAGTCCTCCGCTTCGCAGCTGAAAATGTGCC
CGGGGCCTACTGTGGGCCTAGCCAGGCCTGCTTACGCAGTGCAGTTTCCCATGAATGAT
GCCCAGTCATTATCACATAACCTGTGGCAAGCCAGCAAGATGGCCCTGGTGACAGCAAA
AGAAACTGCACTAGGACCTGAATGTAGATCTCAGTCATGTTCCTTACTAACAGCACGTTT
TGCAACCATGCGTTAAAGAAACATCTGACTCACAACAAAATTTTAAAGGGTTTATTTGA
GTGAAAAGCAATTTATGAATTGGGGAACACCTGACTGAAAGAGCGTTAGTATTCCAGAG
ACAAAACATCAAGTGCAAGTTTTTATTGGGAAAATGTAGAAGCACAATAAAGAAATTAT
TTGATTGGTTAGGATCC

Sequences of Anti-sense：
AAGCTTTAACCAATCAAATAATTTCTTTATTGTGCTTCTACATTTTCCCAATAAAAACTTG
CACTTGATGTTTTGTCTCTGGAATACTAACGCTCTTTCAGTCAGGTGTTCCCCAATTCATA
AATTGCTTTTCACTCAAATAAACCCTTTAAAATTTTGTTGTGAGTCAGATGTTTCTTTAAC
GCATGGTTGCAAAACGTGCTGTTAGTAAGGAACATGACTGAGATCTACATTCAGGTCCT
AGTGCAGTTTCTTTTGCTGTCACCAGGGCCATCTTGCTGGCTTGCCACAGGTTATGTGAT
AATGACTGGGCATCATTCATGGGAAACTGCACTGCGTAAGCAGGCCTGGCTAGGCCCAC
AGTAGGCCCCGGGCACATTTTCAGCTGCGAAGCGGAGGACTAGCAACCGGTGAGGTAG
AAGGAGAACCAAGAGATGGTGGGAGAATGGGAACTGAGCTGAGAGAGCTTCCGGAAGG
TTGCGGTGGCCTAGGGCGATCCACGTCATTGAGAAACGGCGTTAGGCTGATTTCACGGG
GCAGATGACATGGAAGTGCTGCTGAGGAAGACGCTGAGCTCCGAGGTGAAAAGGGATG
AGGACGCCTAAGAAGGATCC

Supplementary Table 2. Sequences of Primers for the RIP assay

	Forward (5’→3’)	Reverse (5’→3’)
LncRNA KLHDC7B-DT	GTTGCTAGTCCTCCGCTTCGC	GCTGGCTTGCCACAGGTTATG
